# Navigating the complex dynamics of anesthesiologists’ professional identity formation in the context of their specialty training program: a phenomenographic perspective

**DOI:** 10.1186/s12909-024-05527-7

**Published:** 2024-05-15

**Authors:** Hanna Chin, Åke Ingerman, Linda Block, Helena Odenstedt Hergès

**Affiliations:** 1 Department of Anaesthesia and Intensive Care, Region Västra Götaland, Sahlgrenska University Hospital, Gothenburg, Sweden; 2 Department of Anesthesiology, Institute of Clinical Sciences, Sahlgrenska Academy, University of Gothenburg, Gothenburg, Sweden; 3 Department of Pedagogical, Curricular and Professional Studies, University of Gothenburg, Gothenburg, Sweden

**Keywords:** Anesthesia, Anesthesiology, Education, Medical, Curriculum, Qualitative research, Phenomenography

## Abstract

**Background:**

A specialty training program is crucial for shaping future specialist doctors, imparting clinical knowledge and skills, and fostering a robust professional identity. This study investigates how anesthesiologists develop their professional identity while navigating unique challenges specific to their specialty. The formation of professional identity in anesthesiology significantly influences doctors’ well-being, teamwork, and ultimately patient care, making it a crucial aspect of anesthesiology education. Utilizing a phenomenographic approach, the research explores the learners’ personal experiences and perspectives of professional identity formation in their specialty training programs, providing valuable insights for enhancing future anesthetic educational programs.

**Method:**

The data for this phenomenographic study were collected through semi-structured interviews with anesthesiology trainees and specialists, guided by open-ended questions. The interviews were conducted at a Swedish university hospital, and participant selection used purposive sampling, providing rich and diverse data for analysis after 15 interviews. Iterative analysis followed the seven-step phenomenographic approach. The research team, comprising qualitative research and anesthesiology education experts, ensured result validity through regular review, discussion, and reflective practices.

**Results:**

The study reveals three fundamental dimensions: ‘Knowledge of Subject Matter,’ ‘Knowledge of Human Relations,’ and ‘Knowledge of Affect.’ These dimensions offer insights into how anesthesiologists comprehend anesthesiology as a profession, navigate interactions with colleagues and patients, and interpret emotional experiences in anesthesiology practice – all crucial elements in the formation of professional identity. The findings could be synthesized and further described by three conceptions: The Outcome-Driven Learner, the Emerging Collaborator, and the Self-Directed Caregiver.

**Conclusion:**

The study uncovers differing learner understandings in the development of anesthesiologists’ professional identity. Varying priorities, values, and role interpretations highlight the shortcomings of a generic, one-size-fits-all educational strategy. By acknowledging and integrating these nuanced learner perspectives, as elucidated in detail in this study, the future of anesthesia education can be improved. This will necessitate a holistic approach, intertwining both natural sciences and humanities studies, focus on tacit knowledge, and flexible teaching strategies, to guarantee thorough professional development, lifelong learning, and resilience.

## Background

A specialty training program plays a vital role in shaping future specialist doctors by fulfilling two important objectives. Firstly, it provides the essential knowledge and skills required for proficient clinical practice. Secondly, it fosters the development of a strong professional identity. Both aspects, are equally important in molding well-rounded and effective practitioners in specialized fields like anesthesiology [1]. In a previous study, we described anesthesiologists’ conceptions of learning anesthesia in the context of a specialty training program [2].This study focuses on anesthesiologists’ conceptions of professional identity formation in the same learning context.

Anesthesiologists often encounter numerous unique challenges in establishing their professional identities. These challenges include earning respect from both patients and peers, despite holding a relatively inconspicuous role within the hospital. Finding the right balance between being a team leader and a service provider simultaneously can be difficult. Additionally, they must nurture various types of working relationships with colleagues, ranging from those with whom they share specific working tasks to more distant connections. Anesthesiologists also grapple with the inherent secrecy and isolation associated with their profession. Furthermore, the perception of anesthesiologists as practitioners of an enigmatic art adds another layer of complexity to their professional identity.

The critical significance of cultivating a robust professional identity in anesthesiology cannot be overstated [3, 4]. It serves as a linchpin that significantly impacts anesthesiologists’ well-being, fostering their self-esteem and personal fulfillment in a relatively stressful specialty [5]. Furthermore, it forms the bedrock upon which effective teamwork, an essential component of anesthetic practice, is built [6]. This trust and cohesion within the healthcare team ultimately enhance patient safety [7]. Finally, a well-defined professional identity helps anesthesiologists make the complex ethical decisions necessary when caring for the most seriously ill patients in the hospital [8].

Professional identity, explored across fields such as psychology, sociology, education, and organizational studies, is complex and multifaceted. In this study, we regard professional identity formation as a socialization process [9]. This process is facilitated by active participation in the professional community, where individuals adopt the collective beliefs, values, attitudes, and behaviors unique to that profession. Creuss has described this concept with a model that provides a comprehensive framework for this socialization process .The model shows how components of professional identity are acquired through various aspects of the curriculum, including the formal, informal, and hidden curriculum [9].

In our study, we aim to uncover the yet understudied socialization process in anesthesia specialty training. By directly engaging with anesthesiology trainees and understanding their experiences ‘on the floor’ we seek to uncover their perceptions of learning anesthesia and discern conceptions of professional identity formation. This approach provides not only a context specific understanding but also uncovers the student perspective rather that an often-expressed teacher or expert perspective.

We have chosen phenomenography approach because it uniquely captures qualitative differences in how individuals understand phenomena in the world, rather than merely describing them [10]. This provides nuanced insights into how individuals interpret and navigate identity formation, offering detailed descriptions and a holistic exploration of influential factors. This phenomenography approach also offers contextual understanding, shedding light on impactful elements such as educational settings and organizational culture. Phenomenography therefore proves beneficial in unraveling poorly understood complex social matters, such as the multifaceted nature of professional identity formation. Ultimately, these insights can inform the development of future anesthetic educational programs.

## Method

### Phenomenography as a research method

Phenomenography, a qualitative research approach, aims to capture the qualitative differences in how people perceive, understand, or conceptualize phenomena in the world. It encompasses a range of phenomena, from specific disciplinary concepts such as reading a text to broader experiences like learning in anesthesiology specialist training programs.

Phenomenographic results consist of categories characterized by distinct ways of conceptualizing a phenomenon, often hierarchically related where the higher-level categories reflect an increasing and more comprehensive, or refined, understanding of the phenomenon, as interpreted by the researcher.

While based on empirical data, they do not reflect specific individuals but rather different collective understandings of the phenomenon. The focus is on understanding variations in participants’ personal experiences and perspectives rather than seeking a single truth [11].

Phenomenography and its results are particularly relevant in educational contexts. They shed light on how individuals perceive what they need to learn and how to learn it, influencing educational engagement and learning practices. Identifying variations in the conceptualization of a phenomenon can highlight areas where students may struggle to learn and inform teaching practices at both local and organizational level [12].

### Data collection

This study collected empirical data by conducting semi-structured interviews with anesthesia specialty trainees and specialists.The interviews were guided by a set of open-ended questions (see Table 1), supplemented with numerous follow-up questions aimed at delving deeper into the interviewees understanding of the phenomenon under investigation. These follow-on questions were designed to elucidate the intended meaning behind the participants’ responses, often prompting for descriptions of concrete experiences. Throughout the interviews, the interviewer refrained from imposing personal viewpoints, leading questions, or introducing new terminology. Participants were given ample time and space to provide detailed and reflective answers. Two pilot interviews were performed to ensure the phenomenon was clearly in focus in the interview and described across a range of aspects by the interviewees as well as hone the researcher’s interview skills. This data was also included in the analysis. All interviews were performed by the primary researcher (HC).

**Table 1:** Interview guide

Open questions:	
•	Describe the specialty trainee program to a doctor new to anesthesiology
•	What is the difference between this postgraduate program and other educational programs you have experienced so far?
•	If you could construct your own training program, what would it look like?
•	Describe a day that was highly educational and a day that was not educational.
•	Describe an excellent and a poor anesthesiologist.

Interviews, which ranged from 20 to 40 min in duration, were audio-recorded using iPhone Voice Memos. The primary researcher (HC) transcribed the recordings verbatim and took field notes to capture any relevant contextual nuances during the interviews. The interviews also served as empirical data in our previous study [2]. However, the present analysis focused on different parts of the interview data, in which professional identity formation came to the fore.

### Setting

The interviews were conducted at a Swedish university hospital offering a five-year anesthesiology and intensive care training program. Prospective specialty trainees typically complete a medical degree followed by an 18-month internship to gain broad clinical experience and obtain a full Swedish medical license. Entry into the competitive anesthesia training program involves applying to individual hospitals, with selection criteria based on academic performance and clinical experience.

The specialty training program includes clinical rotations, mandatory courses, and dedicated time for self-directed study and research, aligning with national health board guidelines and recommendations from the Swedish Anesthesiology and Intensive Care Association. Upon completion, graduates are conferred specialized physician status in anesthesia and intensive care medicine.

### Participant selection

Phenomenography seeks to encompass diverse perspectives on a specific phenomenon. Therefore, this study used purposive sampling to select participants with varied backgrounds, academic abilities, and anesthesia experience. Table 2 shows some of the demographics of participants. After 15 interviews, data saturation occurred, indicating that additional interviews would not capture anymore variation in understanding of the phenomenon. In the context of our study, data saturation refers to reaching a point where the sample size is considered sufficient to provide comprehensive insight and answer the research question with confidence, which denotes substantial information power [13].

**Table 2:** Demographics of participants

	Total	Female	Male
Trainee year 1	6	3	3
Trainee year 2–3	4	2	2
Specialists	5	2	3

### Data analysis

The interview transcripts underwent analysis following the seven-step phenomenographic method outlined by Dahlgren and Fallberg [14]. Table 3 presents in greater detail the steps undertaken in this study.

**Table 3:** Seven steps of analysis in phenomenography for this study

Steps	Data analysis Dahlgren & Fallberg	Data analysis this study
1	Familiarization	All transcripts read by HC; 5 transcripts by HOH, ÅI
2	Condensation	Identifying and coding meaningful units in the transcripts: Coding of 5 transcripts by HC, HOH, ÅI. Then all transcripts by HC
3	Comparison	Comparing the units with similarities and differences: HC with group discussion HC, HOH, ÅI
4	Grouping	Allocating answers expressing similar ways of understanding the phenomenon to the same conceptions: HC with group discussion (HC, HOH, ÅI, LB) External validation by external field experts.
5	Articulating	Capturing the essential meaning of a certain conception. HC & group discussion
6	Labelling	Expressing the core meaning of the conception. HC & group discussion
7	Contrasting	Comparison of conceptions with regard to similarities and differences. HC & group discussion

### Research team and reflexivity

The phenomenographic study followed stringent quality standards, as recommended by Sin [15], and guided by the COREQ checklist for reporting findings. The research team included experts in qualitative research, particularly in phenomenography, and professionals in anesthesia postgraduate education. To ensure result validity, interview findings underwent regular review and discussion in team meetings, reaching a consensus by triangulating insights from the core research team and external experts. The team’s diverse backgrounds bolstered this triangulation process. Reflective practices and critical self-examination were maintained throughout the study to mitigate bias.

## Results

Through iterative analysis, the study unveils three fundamental dimensions that hold significant importance in comprehending the complexities of learning experiences within the anesthetic specialty training program and specifically the development of professional identity.

The first dimension, “Knowledge of Subject Matter,” describes how an anesthesiologist comprehends anesthetic subjects or disciplines differently. The knowledge gained from hospital work, academic studies, or personal learning, shapes their beliefs and values, contributing to the unique facets of their professional identity.

Moving to the second dimension, “Knowledge of Human Relations,” it describes how anesthesiologists view their interactions with colleagues and patients. These understandings not only shape professional identity within anesthesiology but also contributes to the evolving sense of self.

Finally, in the third dimension, “Knowledge of Affect,” variations in individuals’ emotional experiences and their ability to interpret feelings in the context of anesthetic practice were observed. Emotions deeply influence personal preferences, reactions and decision-making, playing a significant role in an anesthesiologist’s identity.

Together, these three dimensions form a framework for examining the intricate interplay between perceived knowledge and identity within the context of an anesthesia training program. The subsequent detailed description provides insights into the variations observed within these three domains, shedding light on the different perspectives and experiences within the anesthesia community.

### The dimension of knowledge of subject matter

This dimension can be described in terms of three qualitatively distinct ways of understanding.

#### Curriculum focused

Here, the knowledge of subject matter is conceived as the pursuit of knowledge through the application of theory and practical skills, adherence to professional standards, and the influence of external motivation. Table 4 shows illustrative excerpts from the interviews.

**Table 4:** Dimension Knowledge of Subject Matter - Curriculum-focused

Interviewee quote	Researcher comment
*“And now that one starts being on call, one thinks, this is not a big problem, I can insert a central line, I can insert arterial lines, I can anesthetize someone. So, one feels confident that one will have a manageable workload all the time.”* (Interview 3, line 104–107)	The focus here is on acquiring aspects of the formal curriculum, including practical and clinical skills.
“(An educational day) *is a day when I have had the opportunity to perform a practical procedure, either an intubation or a central venous catheter, once or twice.”* (Interview 1, line 93)	An educational day is defined as a day during which you acquire a series of isolated practical skills.
*“If you are going to perform a lumbar puncture, for example, you should read about it and understand the indications. You should understand how to do it practically… you should understand the anatomy, when to puncture. It’s not something you can learn just by reading, and you learn much more by doing it practically”* (Interview 4, line 82–84)	Here, the emphasis is on learning practical procedures, i.e., the formal curriculum.

#### Educational Multivalence

Here, the conception of knowledge of subject matter centers on the simultaneous embrace of diverse perspectives, shaped by external influences. It involves prioritizing the ideas, norms, and beliefs of the surrounding systems. Table 5 presents representative quotes extracted from the interviews.

**Table 5:** Dimension Knowledge of Subject Matter - Educational multivalence

Interviewee quote	Researcher comment
*“Partly to reflect on my own, but also to leverage the experiences and colleagues that are available and discuss with them… Initially, I thought that some colleagues were a bit paranoid about certain things, but then I thought this: if those colleagues are significantly more experienced than I am, and I’ve never been in that situation before, it’s probably good to be paranoid and err on the cautious side. It’s easy for me to think, having only read about it in books, that if something happens, you just do this, this, and that. However, if those who have experienced it many times and respect it, then I must have even more respect for it.”* (Interview 3, line 202–209)	Here, there is an awareness of the significance of practical wisdom, respect for experience, and the value of learning from others within their professional field
*“So, then I heard this person discussing with other colleagues, had done a lot of research on their own, planned to use these medications, had prepared beforehand, seen the patient, talked to the patient’s relatives who were present, and then just nothing happened because it was so well-prepared”* (Interview 2, line 109–112)	Here, anesthesiology knowledge is demonstrated to arise from various sources, including interactions with people around them.

**Autonomous Mastery of Learning.** Here, the knowledge of subject matter is conceived as self-crafted principles, gracefully adapting to and embracing complexity. Table 6 provides examples in the form of excerpts from the conducted interviews.

**Table 6:** Dimension Knowledge of Subject Matter - Autonomous mastery of learning

Interviewee quote	Researcher comment
*“We have, of course, the theoretical knowledge, measuring it is the easier part. The other part is hopefully through a well-functioning workplace where someone can provide you with their reflections on how you’ve handled different patient cases. Having discussions with you, debating, and considering how these things could be different. Also, organized discussions where it’s clear that you can identify if someone is having difficulty meeting these collaboration goals. I’m thinking that several specialists sit down and compare their experiences, and that’s an important aspect too. If it doesn’t happen in an organized context, it tends to happen on the sidelines in a non-constructive manner*.” (Interview 13, line 20–27)	Here, all aspects of learning are considered in a mature, self-reflective, critical, and confident manner.
*“So, I’ve noticed with some of my colleagues, at least, that they have sort of evolved and started to see things in a different way. They think about different things and focus on different aspects. They are a bit more flexible and don’t just get stuck in their thoughts and ideas.”* (Interview 11, line 224–226)	Here there is an understanding for the need to adapt flexibly to situations.

### The dimension of knowledge of human relations

This dimension can be described in terms of three qualitatively distinct ways of understanding.

#### Individual-centric

Here the focus is primarily on personal performance, accomplishments, and self-validation, with less attention to patients and work colleagues. It shows the importance in striking a balance between external opinions and self-interest while fostering transactional relationships for personal achievement. Table 7 are example quotes form the interviews.

**Table 7:** Dimension Knowledge of Human Relations - Individual-centric

Interviewee quote	Researcher comment
*“(an excellent anesthesiologist) should be able to train and work with less experienced colleagues, helping them manage the job, knowing when to step in to avoid harm to the patient while allowing their apprentices and coworkers to take as much responsibility as possible. They should be able to provide constructive feedback, understand the risks associated with their work, and approach their duties with an ethical mindset.”* (Interview 1, line 144–148).	Here, the person describes an excellent anesthesiologist as someone who can assist and guide through being a good teacher, serving as an example within a transactional relationship.
*“For example, if you anesthetize something complicated, and you work for half an hour in the morning, and everything went quite smoothly, like a Whipple or something. You place your central venous catheter, you insert your arterial line, and then you administer anesthesia. After that, you’re in the operating room, checking a few times, but not much else happens; you aren’t really challenged.”* (Interview 3, line 128–131)	Here, the person is focused on personal performance and learning satisfaction, with the patient being somewhat invisible.
(a difficult epidural in the postoperative ward) “*There was a lot of room for me to practice. I felt safe and calm, and I thought that was very good.”* (Interview 1, line 156–157)	Here, there is a focus on personal learning satisfaction.

#### Community aware

This understanding acknowledges the influence of team members and patients and emphasizes the impact on the people in one’s surroundings. It gives importance to external perception and validation and seeks role models for shaping one’s own identity. Table 8 provides illustrative excerpts from the interviews.

**Table 8:** Dimension Knowledge of Human Relations - Community aware

Interviewee quote	Researcher comment
*“I never feel that I’m standing there pondering and coming up with things on my own without always discussing my thoughts with someone, going through what I intend to do with a patient with either the nurse standing beside me, bouncing around some ideas, or with another doctor who has more knowledge than I do, seeing what is reasonable. Then you get an exchange in that moment.”* (Interview 2, line 20–24)	This quote reflects a collaborative and dialogical approach to decision-making and reflective practice. The participant emphasizes the importance of discussing thoughts and intentions with others.
*“A good anesthesiologist should take the leadership role seriously. You don’t work alone. You work in a team with nurses and nursing assistants. You have the opportunity to discuss and justify your decisions.”* (Interview 11, line 189–190)	This quote emphasizes the collaborative nature of healthcare and underscores the importance of teamwork.
*“It’s important to be friendly because teamwork is crucial, as well as being good at communication and treating patients kindly, as one can achieve a lot simply by calming them down when meeting them”* (Interview 3, line 167–170 )	Here there is an emphasis on teamwork and effective communication, showing the significance of interpersonal skills in the medical field.

#### Empathetic-centered

This understanding prioritizes positive and respectful interactions with others, both in personal and professional settings, while demonstrating a deep understanding of how one’s actions impact the broader community. This person operates independently of external validation and remains grounded in professional values. Table 9 shows the interviewee quotes.

**Table 9:** Dimension Knowledge of Human Relations - Empathetic-centered

Interviewee quote	Researcher comment
*“(Bad anesthesiologist) When you think this patient is just going to be operated on, not caring about who they are or what their name is. Yes, when you don’t have a really good communication with the nurse, and you don’t really want to be involved.”* (Interview 5, line 213–215)	This quote reveals a negative view of anesthesiologists who lack empathy and fail to establish effective communication, both with patients and nurses.
*“Through conversations, listening, and taking the time. That’s how you solve it, and also pay a lot of attention to other categories of staff who may be closer to the patient, trying to pick up signals if something is not right, if something is not as it should be. And to find solutions when there are conflicts - we want what’s best for the patient, and that’s the recurring theme, that it should always go well for the patient.”* (Interview 15, line 473–478)	Here there is an emphasis on the importance of being attuned to signals from various staff members, with a shared commitment to ensuring the best outcomes for the patient.
*“But I can think that you learn from the discussion with trainees and supporting them; I also learn from that. They have later and better education. They have other tools than what I/we have learned. So, I learn from the discussion. It’s still a lot from the conversation with them, but from the other direction.”* (Interview 12, line 82–85)	This insight underscores the importance of fostering respectful interactions with all colleagues, irrespective of their positions.

### The dimension of knowledge of Affect

This dimension can be described in terms of three qualitatively distinct ways of understanding.

#### Emotional dominance

Here there is he capacity for emotions to prevail over rational thinking, coupled with less focus on self-reflection. Table 10 shows the excerpts from the interviews.

**Table 10:** Dimension Knowledge of Affect - Emotional dominance

Interviewee quote	Researcher comment
*“I have support and get to see a lot and feel safe, then I think I’ve had a good day. I had been alone; I wouldn’t have been able to manage.”* (Interview 4, line 111–113)	This quote expresses the importance of support and a sense of safety for the individual in evaluating a good day.
*“It’s challenging to join as a newcomer; you’re essentially the weakest link in the team. It doesn’t take just a month to fit in, be ‘cool,’ and start performing well; you feel like a burden all the time. I believe it varies how different individuals on the nursing side perceive you. Eight out of ten have been very friendly and caring, but then you sense that there are some people, for some reason, who consider you a burden and think you’re in the way”* (Interview 2, line 87–95)	The quote highlights the challenges of being a newcomer underscoring the importance of a supportive and welcoming work environment.
*“It has to do with how colleagues treat you. You feel like you’re in the way and not as good as your colleagues and other ST doctor colleagues. It takes you a little longer and you get stressed out by time pressure and not feeling adequate. On those days, you go home feeling like you haven’t done anything good.”* (Interview 4, line 123–126)	The quote underscores the impact of colleagues’ treatment on professional well-being, potentially leading to feelings of inadequacy and stress.

#### Reflective awareness

In this state emotions are managed effectively, but with a limited sense of independent self-identity. Table 11 provides illustrative interviewee quotes.

**Table 11:** Dimension Knowledge of Affect - Reflective awareness

Interviewee quote	Researcher comment
“*One must constantly reflect on what one is doing and continually strive for improvement. In those instances when it doesn’t work as it should, one must be self-critical*. ” (Interview 15, lines 362–364)	The statement reflects a proactive approach to professional development including self-reflection and a willingness to learn from challenges.
*“Well, yes, no, but I believe that personal feedback is crucial in understanding how one is perceived in your room, so to speak. It can be quite challenging to receive it, as it is sensitive, but I think it’s what opens one’s eyes very often”* (Interview 8, line 249–250)	Here there is an understanding that feedback is crucial for self-reflection.

#### Emotional proficiency

Here there is ability to author one’s desires and life direction, maintaining a self-sustaining identity. Table 12 shows the quotes from the interviews.

**Table 12:** Dimension Knowledge of Affect - Emotional proficiency

Interviewee quote	Researcher comment
*“That one allows their own reactions to the difficult aspects we’re exposed to, to influence the treatment or actions, or how one leads the team. You lead a team every time. One must be quite aware of how it affects others in the team, how one behaves. If one is not conscious of their own role in this, then they become a rather poor anesthesiologist, regardless of whether they are skilled or not.”* (Interview 12, line 112–116 )	Here highlights the need for conscious awareness of one’s behavior and its effects on the team, suggesting that being a skilled anesthesiologist goes beyond technical expertise and requires a mindful approach to interpersonal dynamics and leadership.
*“I believe that it helps to be a bit humble and supportive both when working with colleagues who may need assistance and also when working with many other specialties. When you’re down in the emergency room for consultations or with other consultants, there are many who have great respect for your specialty. I think the ability to invite others into discussions and make them feel involved and seen is crucial so that they don’t feel overridden or ignored, as I have experienced.”* (Interview 8, line 207–211)	The focus is on creating a collaborative environment that values diverse expertise and fosters a sense of involvement and respect among professionals.
*“I often think about my grandmother and grandfather, or even about my mom. I wouldn’t want to expose my mom to this, or my grandmother to this. It’s like, where should one draw the line, so as not to completely… I don’t know, it’s real, but emotionally, and what are we doing right now, and should we continue, or should we not do more, like, what should one do about it, what, how should one treat one’s patient.”* (Interview 15, line 201–206)	This statement reveals a complex interplay of reflective thinking, emotional considerations, ethical dilemmas, and uncertainty.

### Synthesized dimensions: an Integrated Perspective

The dimensions described above can be summarized by three conceptions denoted as the Outcome-Driven Learner, the Emerging Collaborator, and the Self-Directed Caregiver, as presented in an outcome space (see Table 13). It is important to note that these results depict variations in collective understandings rather than individual understandings. This implies that an individual’s descriptions of professional identity formation could be represented in all three categories, depending on the context and the focus at hand. By framing the results in this manner, the aim is to foster a deeper understanding of the multifaceted nature of professional identity formation, acknowledging the fluidity and complexity inherent in individuals’ experiences and perspectives. This approach encourages a nuanced exploration of the driving forces and values that shape professional identities, facilitating richer discussions and insights into the dynamics of professional development within various contexts and fields. The intention is therefore not to categorize or label individuals per se.

**Table 13:** Outcome space

	Knowledge of Subject Matter	Knowledge of Human Relations	Knowledge of Affect
**The Outcome-driven Learner**	Curriculum focused	Individual-Centric Approach	Emotional dominance
**The Emerging Collaborator**	Educational multivalence	Community aware	Reflective awareness
**The Self-directed Caregiver**	Autonomous mastery of learning	Empathetic centered	Emotional proficiency

**Tables**

### The outcome-driven learner

This individual demonstrates a strong interest in pursuing a career in the anesthesiology profession and is motivated to acquire the necessary skills, primarily through the formal curriculum. They emphasize personal achievement and recognition, viewing professionalism as excelling in knowledge application and adhering to external regulations. Their working relationships tend to have a transactional nature. Adherence to rules is often influenced by external rewards rather than purely personal beliefs.

They sometimes experience a sense of being an outsider in their professional environment, leading to stress as they perceive a lack of respect, value, and full integration.

### The emerging collaborator

This individual is an active member of a professional team, working to establish their role within it. They look for role models to learn from and are beginning to experience a sense of belonging, although they may not feel completely at ease yet. External validation plays a significant role in shaping their self-perception, as they are still in the process of developing a fully independent identity. Their stress levels have reduced compared to their past experiences.

Their focus has shifted towards being a collaborative team member and adhering to external societal and professional standards. They can consider various viewpoints and are willing to put collective interests ahead of their own. They are characterized by their idealism, capacity for self-reflection, and a commitment to prioritizing ethical and morally sound actions.

### The self-directed caregiver

This individual has a strong sense of self that is independent of external influences such as others, relationships, or their environment. They have developed an internal sense of purpose and take charge of shaping their own life path.

They place a high priority on fostering interpersonal connections, with a particular focus on understanding and nurturing relationships. Their approach to learning is marked by autonomous mastery, as they create their own principles for learning and adeptly adapt to complex situations. Moreover, they demonstrate emotional control, effectively managing their desires and the direction of their life.

### Discussion and conclusion

The results provide coherent descriptions of the intricate and multifaceted process of professional identity formation within the field of anesthesiology. These insights can be effectively encapsulated within a framework, offering a comprehensive understanding of the subject. The implications of these findings extend to the enhancement of future specialty training programs for anesthesiologists, as elaborated upon below.

### Different understandings

Different perspectives on professional identity formation in anesthesiology have been identified in this study. This variation in anesthesiologists’ understanding of learning within a specialty training program aligns with findings from our previous study [2] and are supported by other researchers [16–18]. In general, there is a developmental trajectory in understanding tied to the years of experience in anesthesiology, although this is not entirely predictable.

The differences in understanding professional identity formation encompass various priorities, values, and role interpretations. For example, while some emphasize personal achievements and compliance with external rules, others prioritize personal purpose, interpersonal connections, and autonomous learning while managing emotions.

This diversity suggests that there isn’t a single approach that fits everyone when it comes to developing professional identities in anesthesiology training programs. Recognizing this, it’s clear that a personalized approach is essential to meet the unique needs and preferences of each individual, which might also change over time. To effectively tailor the training program, it’s important to openly discuss and understand these various perspectives, benefiting both the learners and educational supervisors.

### Impact on well-being

The trainees’ understanding of emotions, including their emotional intelligence and self-reflection abilities, is crucial in shaping their professional identity. We have found a spectrum of understanding ranging from instances where emotions overshadow rational thinking with minimal self-reflection to the ability to independently shape aspirations and maintain a strong self-identity. These differences can significantly impact an individual’s well-being [19].

Globally, there is a growing recognition of the importance of learner well-being in improving learning and performance of anesthesiologists [20, 21]. Affect is a central part to patient safety [8]. Therefore, it’s essential to incorporate this aspect into improvement of future specialty training programs. Two areas of potential improvements are:

Firstly, integrating personal support mechanisms such as emotional intelligence training and self-awareness programs could be instrumental in helping anesthesiologists navigate the emotional challenges inherent in their profession. This is especially pertinent for “Outcome-driven Learner” who may struggle with initial self-doubt and feelings of inadequacy.

Secondly, creating a supportive social environment within training programs is crucial. This includes fostering a sense of belonging [22], offering mentorship, and addressing factors that can worsen negative emotions [21]. This is significant because studies indicate that a lack of support at work is strongly associated with burnout, emphasizing the need for organizational support [20].

### View on interpersonal relationships

This study shows different understandings when it comes to personal interests and relationships with others. Some anesthesiologists prioritize personal achievements and recognition, while others value teamwork and focus on connections with others.

Firstly, this highlights the importance of balancing individual goals with group responsibilities. This balance is crucial for maintaining a work environment that is both cooperative and efficient, creating an optimal learning environment [23].

Secondly, it emphasizes the need to build a supportive and collaborative culture in anesthesiology practice. This culture is essential for fostering a shared dedication to patient care [24]. Emphasizing teamwork not only improves the quality and safety of patient care but also helps create a cooperative and unified professional community.

### Patient-centered care

We have found that the professional identity formation involves a range of attitudes towards patients. In some instances, the patient seems almost invisible to the anesthesiologist, while in other cases, the patient takes center stage.

A study by Aagard and colleagues [25] highlighted a similar concern among anesthetic nursers. They found that nurses may sometimes focus too much on technical procedures and overlook the emotional and relational aspects of patient care. This emphasis on efficiency and specialization could lead to lack of attention to the human side of nursing care, particularly in interactions with patients.

Given that a fundamental tenet of professionalism is “prioritizing the patient’s interest over the provider’s interest” [17], it becomes crucial to address this dimension during specialty training programs for both anesthetic nurses and anesthesiologists. Recognizing and rectifying the potential oversight of patient-centric care in the face of highly practical specialty is essential for fostering a comprehensive and patient-focused approach within the anesthesia profession.

### Study limitations and future studies

Our study, conducted at a single Swedish university hospital, offers valuable insights into professional identity formation among anesthesiology trainees. However, its applicability to other contexts may be limited by differences in anesthesiology training programs, cultural factors, and healthcare systems. Also, while phenomenography enabled us to capture diverse perceptions of professional identity, it is important to acknowledge that it may not have fully explored the essence of professional identity formation of anesthesiologists in depth.

Furthermore, the cross-sectional design of our study may not have adequately captured the longitudinal dynamics of identity development. Therefore, there is a need for further investigation to determine whether the majority of learners progress through the understanding spectrum during their training program, and how some individuals may attain certain understandings earlier than others. This exploration should consider both individual traits and external influences such as learning activities on the progression of professional identity.

Additionally, it is important to investigate how the presence of educators with different understandings may impact the learning environment and educational outcomes.

Furthermore, it would be beneficial to explore how variations in professional identity understandings among anesthesiologists may influence patient outcomes, taking into account the perspectives of patients themselves. This would provide a more comprehensive understanding of the relationship between professional identity and patient care.

## Conclusion

In conclusion, when considering a specialty training program for anesthesiologists, a holistic approach to professional identity formation must transcend the confines of natural sciences and extend to include humanities, acknowledging the multidimensional nature of this professional developmental process [18]. The incorporation of tacit knowledge learning into formal teaching is paramount, necessitating a focused effort on areas marked by variation [19]. Effective teaching strategies should prioritize activities that enhance the learning process, empowering learners to construct their own goals, receive constructive feedback, engage in reflection, and consolidate their evolving understanding. This emphasis on the learning process, rather than rigid and prescriptive content, is pivotal in nurturing adaptable and resilient professionals.

Recognizing the significance of interventions becomes crucial, particularly when challenges arise in professional identity formation. Adopting a comprehensive perspective ensures a robust foundation for lifelong learning and sustained professional growth.

## Data Availability

The datasets generated and/or analyzed during the current study are not publicly available due to promised anonymity of the participants but are available from the corresponding author on reasonable request and with permission of the participants in question.
